# Renal Anemia Control in Lithuania: Influence of Local Conditions and Local Guidelines

**DOI:** 10.1155/2013/260915

**Published:** 2013-12-03

**Authors:** Edita Ziginskiene, Vytautas Kuzminskis, Kristina Petruliene, Ruta Vaiciuniene, Asta Stankuviene, Inga Arune Bumblyte

**Affiliations:** ^1^Department of Nephrology, Medical Academy, Lithuanian University of Health Sciences, Mickeviciaus str. 9, LT-44307 Kaunas, Lithuania; ^2^Nephrological Clinic, Hospital of Lithuanian University of Health Sciences Kaunas Clinics, Eiveniu str. 2, LT-50009 Kaunas, Lithuania

## Abstract

Erythropoietin stimulating agents had a long haul in Lithuania—we had no epoetin till 1994 and there was no intravenous iron in 2001–2004. The aim of this study was to assess the changes of renal anemia control in hemodialysis patients from early independence of Lithuania till nowadays and to evaluate the link of anemia with hospitalization rates and survival and hemoglobin variability in association with mortality. In December of each year since 1996 all hemodialysis centers have been visited and data has been collected using special questionnaires. The history of renal anemia control in Lithuania was complicated; however, a significant improvement was achieved: 54.7% of hemodialysis patients reached the target hemoglobin; all patients have a possibility of treatment with epoetin and intravenous iron. The involuntary experiment with an intravenous iron occurred in Lithuania because of economic reasons and confirmed the significant role of intravenous iron in the management of renal anemia. Hemoglobin below 100 g/L was associated with a 2.5-fold increase in relative risk of death and 1.7-fold increase in relative risk of hospitalization in Lithuanian hemodialysis patients. Although hemoglobin variability was common in Lithuanian hemodialysis patients, we did not find the association between hemoglobin variability and all-cause mortality in our study.

## 1. Introduction

Lithuania is a country in Northern Europe, the largest of the three Baltic States. It is situated along the southeastern shore of the Baltic Sea with a territory of 65 200 km^2^ and a population of 3 million inhabitants. Starting in 1940, Lithuania was occupied by the Soviet Union. On March 11, 1990, the year before the breakup of the Soviet Union, Lithuania became the first Soviet republic to declare independence. In the early period of independence (1991–1993) hemodialysis (HD) was only acetate and available only for recipients waiting for transplantation. There were old HD machines, no water treatment, lack of nephrology literature in English and no competent training in nephrology. This was followed by tremendous progress in renal replacement therapy later on: full renovation and expansion of HD service, start of peritoneal dialysis, establishment of a second center of kidney transplantation, development of a Western model of nephrology with the help of European Renal Association-European Dialysis and Transplant Association (ERA-EDTA) and International Society of Nephrology (ISN).

The introduction of erythropoiesis stimulating agents (ESAs) has changed the management of renal anemia, leading to substantial reduction in the blood transfusion requirements, improvement in energy and physical function and improvement in health-related quality of life. However introduction of ESAs had a long haul in Lithuania: we did not have epoetin until 1994 and there were no intravenous iron in the period of 2001–2004 and very strict limitations by Lithuanian Ministry of Health for the prescription of epoetin. The Lithuanian anemia management guidelines were revised only in August 2011 and correspond to European Renal Best Practice (ERBP) statements today, but, until then, hemoglobin (Hb) target range of 100–105 g/L was recommended. Maintaining Hb levels within such a narrow target range was a challenge in our clinical practice, so Hb variability was highly prevalent in our dialysis patients.

First data about control of renal anemia in HD patients in Lithuania were published in 2003 [1]. Authors presented relationship between lethality of HD patients and renal anemia control. They concluded that adequate HD procedures and a good management of HD patients decreased requirement of erythropoietin doses for renal anemia treatment. The aim of this study is

1. to analyse the changes of renal anemia control in HD patients depending on local protocols from early independence of Lithuania till nowadays;
2. to evaluate the link of anemia with hospitalization rate and survival;
3. to evaluate Hb variability in association with mortality.

## 2. Materials and Methods

In the absence of official Renal Registry in Lithuania, in December of each year starting from 1996, all HD centers of the country have been visited and data has been collected using special paper questionnaires. Information about the number of patients and HD stations, demographic characteristics, etiology of end stage renal disease (ESRD), data about dialysis quality, blood tests, and the medicines used have been obtained. Changes of renal anemia control in HD patients depending on local protocols were evaluated from early independence of Lithuania till nowadays.

Influence of anemia on hospitalization rate was evaluated in a prospective study performed in 2002–2006. We investigated 559 patients from Kaunas region of Lithuania. The Kaunas region accounts for 12% of the Lithuanian territory and for 20% of the population. During the study 27% of all Lithuanian ESRD patients were hemodialysed in Kaunas region. Kaunas HD patients were representative of overall Lithuanian HD population: a comparative analysis using all Lithuanian data showed no statistically significant differences in age, gender, primary cause of ESRD, and hospitalization rate. Patients were followed prospectively 12 month for hospitalization rate, length of hospital stay, and causes of hospitalization.

Using data, collected at annual visits to HD centres, study on mortality of HD patients was performed. All patients who started chronic HD due to ESRD in Lithuania, between January 1, 1998 and December 31, 2005, were enrolled in our study. Outcomes and mortality and survival rates were analysed in the study.

Hb variability in HD patients was evaluated in another single-center, retrospective study (*n* = 100). This study was conducted in Lithuanian University of Health Sciences, Department of Nephrology. The study started on January 1, 2011 and the follow-up included 12 months till December 31, 2011. Serum Hb concentrations and ESA doses were measured each month for each patient. Monthly Hb values were categorized as low (L; <100 g/L), intermediate (I; 100–105 g/L), and high (H; >105 g/L), according to our local renal anemia management algorithm at that time, which defined a target range of Hb 100–105 g/L. Then a six-group classification system (according to [20]) was used based on the lowest and highest Hb categories seen during the six-month observation period (01/2011–06/2011): low-low (LL)—consistently low; intermediate-intermediate (II)—consistently within the target range; high-high (HH)—consistently high; low-intermediate (LI)—all six months with low or target range Hb values; intermediate-high (IH)—all six months with high or target range Hb values, and low-high (LH)—fluctuation of low, high, and target range Hb values within six-month period. The association of Hb levels and Hb variability with mortality was evaluated.

### 2.1. Statistical Analysis

For the statistical analysis we used Statistical Package for Social Science, version 20.0. Variables included in the study were expressed as percentages or position (mean, median) and dispersion parameters as appropriate for the type of variable. For evaluation of continuous variables the statistical mean and standard deviation were used. Kolmogorov-Smirnov statistics were used to evaluate sample normality distribution. Comparison between groups was performed using the Student's *t* test, chi-square test, and Mann-Whitney *U* test. Spearman's rank correlation coefficient was used to evaluate relationship between sets of data. The cumulative survival rate was estimated using the Kaplan-Meier method. The event of interest was death. Univariate Cox proportional hazards analysis was used to select variables significantly associated with the risk of death; then these variables were included in multivariate Cox proportional hazards models. Relative risk of hospitalization according to laboratory tests was estimated using Cox regression analysis model. Significant values were considered when *P* < 0.05.

## 3. Results and Discussion

### 3.1. Development of HD Service and Control of Anemia in Lithuania during 1996–2010 Period

Tremendous changes were observed in HD service of Lithuania during this period. There was an increase in number of HD centres (from 17 to 61) and HD stations (from 25 p.m.p. to 201 p.m.p.) in 1996–2010. The prevalence of HD patients increased from 60 p.m.p. in 1996 to 467 p.m.p. in 2010 and the incidence rate of HD patients increased from 54.3 p.m.p. in 1997 to 115 p.m.p. in 2010. The mean age of the prevalent HD patients increased from 47.2 ± 16.1 to 61.1 ± 15.4 (minimum 13, maximum 96, median 64) years. 84.7% of HD patients was dialysed 12 and more hours per week in 2010, versus 30.8% in 1996, *P* < 0.001. Mean Kt/V was 1.34 ± 0.25 in 2010 versus 0.81 ± 0.53 in 1999, *P* < 0.001. Improvement of the quality of HD was associated with improvement of anemia control during the period of 1997–2010. The mean Hb concentration increased from 92 ± 15.4 g/L to 107 ± 13.6 g/L, and the percentage of patients with Hb >100 g/L increased from 27.5% in 1997 to 68.2% in 2010 (Table 1). These changes were statistically significant during the first years of observation (Table 1). The target Hb level in patients on chronic HD was between 100 g/L and 105 g/L during the study according to our national algorithm for the management of anemia in Lithuania (it was introduced on 2000). The target of Hb is debated to this day. K/DOQI guidelines [2] and European Best Practice Guidelines [3] have recommended Hb target of 110 to 120 g/L and >110 g/L, respectively. 2012 KDIGO guidelines suggested limitation of the upper Hb level to ≤115 g/L [4]. So Hb 107 g/L was sufficient according to national and KDIGO guidelines in 2010, but it was too low as compared with other recommendations. According to results of The dialysis outcomes and practice patterns study (DOPPS), the same mean Hb of prevalent HD patients as in Lithuania was observed only in Japan (104 g/L in DOPPS III) [5]. Japanese Society for Dialysis Therapy recommended that a Hb level of 110–120 g/L at the first dialysis session in week is desirable in relatively young patients [6]. While it holds that the Hb level of the Japanese population seemed to be low when compared with that of the European and American populations, the mean Hb of other countries in DOPPS III was 115–120 g/L [5].

In 2010 76.6% of patients received epoetin in Lithuania. This percentage was low as compared to results of DOPPS study in 2010: lowest percentage was observed in Japan (87.3%) and Austria (87.7%) and ranged from 89.2% (France) till 96% (Belgium) in other countries [7]. In Lithuania 51.5% of HD patients was treated with epoetin beta, and 43.3% with epoetin alfa in 2005. Treatment with darbepoetin alfa was started in 2005 in Lithuania, so only 5.2% of patients received this medication. Increase of long-acting ESA usage was observed during 2005–2010. Percentage of patients treated with darbepoetin increased till 48.4% in 2010 in Lithuania as compared to 45.8% of patients in UK, 50.7% in Japan, and only 6.1% in USA [7]. Mircera is registered but not reimbursed in Lithuania so our HD patients have a possibility of this treatment only in frames of ongoing clinical trial. 25% of HD patients in France and 15.5% in Belgium were receiving Mircera in DOPPS study in 2010 [7].

### 3.2. The Role of Intravenous Iron (Experience of Lithuania)

**Figure 1:**
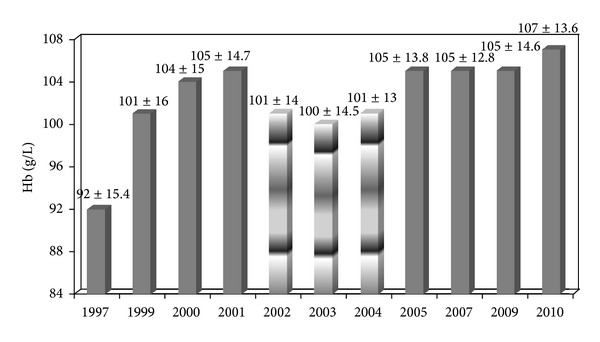
Changes of mean hemoglobin concentration of hemodialysis patients due to influence of national algorithm and deficiency of iron in 1997–2010.

The oral route of iron administration was popular in Lithuania before 1997. Only 7.5% of patients received intravenous iron. After the increased use of intravenous iron in the year 2000 the mean Hb concentration increased significantly without serious changes in the doses of epoetin (Hb 104 ± 15 g/L in 2000 versus 101 ± 16 g/L in 1999, *P* < 0.05, Table 1). However in the period of 2001–2005 intravenous iron was poorly available in Lithuania. The percentage of patients receiving intravenous iron sharply decreased till 20.9% in 2001, and the Hb concentrations did not change at the expense of significant increase of the epoetin dose in this year (9336 ± 3571 U/week versus 7092 ± 3424 U/week in 2000, *P* < 0.001). Our results coincided with the data of other studies confirming importance of the intravenous route of iron administration in CKD HD patients as compared to oral administration [8, 9]. Intravenous iron administration led to a greater increase in Hb concentration, a lower ESA dose, or both in most studies [4]. Limitations to the prescription of epoetin were introduced by Lithuanian Ministry of Health at the same time with unavailable intravenous iron. This was followed by worsening of the control of renal anemia in 2002. According to this new algorithm target Hb was 100–105 g/L for HD patients and maximum weekly dose of epoetin was 20000 IU. The mean Hb concentration decreased to 101 ± 14 g/L, the percentage of patients with Hb >100 g/L decreased to 51.8%, the percentage of HD patients receiving epoetin decreased to 88.8%, and the mean weekly dose of epoetin decreased to 7145 ± 3882 U, *P* < 0.001 (Table 1). The rules of renal anemia treatment were very strict in Lithuania, so it was difficult to keep higher Hb concentration. Fortunately usage of intravenous iron (iron dextran and iron sucrose) was restarted in 2005 and situation of anemia control improved. Hb concentration increased to 105 ± 13.8 g/L (*P* < 0.001), the percentage of patients with Hb >100 g/L increased to 65.1%, the percentage of HD patients receiving epoetin decreased to 84%, and the mean weekly dose of epoetin decreased from 8121 ± 6243 U in 2004 to 6768 ± 4372 U in 2005 (Table 2). All these changes were statistically significant. The changes of mean Hb due to influence of national algorithm and deficiency of iron are presented in Figure 1. Insufficiency of iron increased between 2002 and 2004, and percentage of patients with ferritin <100 mcg/L decreased till 18.5% in 2005 (*P* < 0.001, Table 2). It is true to say that Lithuania had involuntary experiment to show influence of intravenous iron for the treatment of renal anemia. It is a pity that this experiment was very expensive as it lasted four years and all patients were involved.

### 3.3. Influence of Anemia on Hospitalization Rate in HD Patients from Kaunas Region of Lithuania

**Figure 2:**
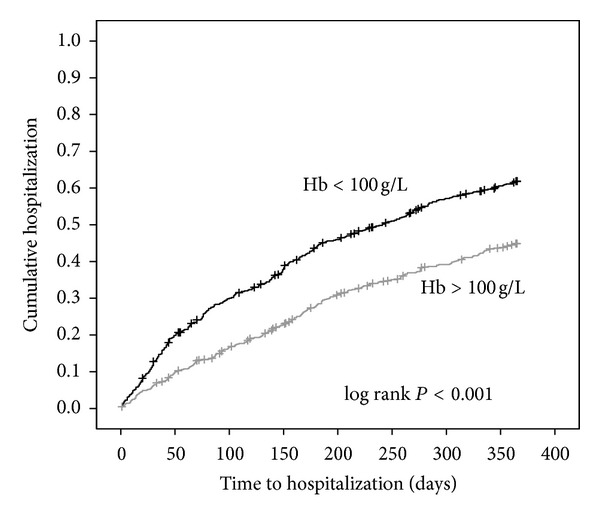
Relation between hemoglobin level and hospitalization in Lithuanian hemodialysis patients.

There is no unified opinion about the influence of anemia to hospitalization rate of HD patients. Big retrospective study of dialysis patients showed that higher concentration of Hb associated with lower rate of hospitalization [10]. DOPPS study (data from 5 European countries) showed that higher Hb concentrations were associated with decreased relative risk of hospitalization: patients with Hb <100 g/L were 29% more likely to be hospitalized than patients with Hb 110–120 g/L [11]. But in prospective randomized trials hospitalization rate did not differ between groups of lower and higher Hb [12]. There were no Lithuanian data about relationship between hospitalization of HD patients and anemia till our study. Relative risk of hospitalization was estimated using Cox regression evaluating time to first hospitalization. Multivariate Cox regression model revealed that relative risk for hospitalization decreased by 0.98 for every 1 g/L rise of Hb (adjusted to age, sex, comorbid conditions, albumin, urea and phosphorus concentrations interdialytic weight gain, nonadherence to medications, systolic blood pressure before and dialysis, disability status). Cutoff value for Hb was <100 g/L: relative hospitalization risk increased by 1.7 (95% CI 1.4–1.95, *P* < 0.001) in patients with Hb <100 g/L (Figure 2).

### 3.4. Association of Anemia with Mortality in HD Patients in Lithuania

Annual data collection allows us to analyse associations between anemia and mortality in incident HD patients in Lithuania in 1998–2005. Analysis revealed that the mean Hb value of all these patients was 101.28 ± 12.59 g/L; in males it was higher than in females (102.34 ± 12.52 g/L versus 100.01 ± 12.56 g/L, *P* < 0.001) and did not differ comparing different age groups and primary renal disease groups.

Multivariate Cox proportional hazards analysis revealed that anemia was an independent risk factor of death (RR  =  0.952, 95% CI 0.945–0.959, *P* < 0.001). Relative risk of mortality was 5% lower for every 1 g/L greater Hb concentration used as continuous variable and adjusted for age, sex, and primary kidney disease.

As shown in Table 3, the relationship of Hb level with mortality varied across different categories of Hb concentrations.

Patients with Hb level of 100 to 105 g/L were selected as the reference group, according to national algorithm for the management of anemia in Lithuania from 2002 (the target Hb level in patients on chronic HD was between 100 g/L and 105 g/L). The Hb concentration below 100 g/L was associated with a 2.5-fold increased relative risk of death. Hb levels of >106 g/L were not associated with a lower risk of death. For Hb concentrations ≥130 g/L, a trend towards higher mortality risk was observed (RR  =  2.356, 95% CI 0.953–5.822, *P* = 0.063), but it did not reach statistical significance. Anemia is associated with an increased risk of morbidity and mortality principally due to cardiac disease and stroke [13, 14]. Hb concentration <100 g/L is independent risk factor of cardiovascular diseases for dialysis patients [15]. DOPPS study showed that higher Hb concentrations were associated with decreased relative risk for mortality [11]. On the other hand, clinical trials showed that maintenance of Hb levels above 130 g/L may be associated with increased morbidity and mortality in dialysis. A recent meta-analysis indicated increased mortality at higher Hb target [12]. A trend towards a higher mortality risk was observed for patients with Hb concentrations >130 g/L in our study.

### 3.5. Hemoglobin Variability in Lithuanian HD Patients

Since the introduction of ESA, most of the clinical trials with ESA therapy have focused on Hb targets in CKD patients; however, there is a shortage of clinical trials studying the optimal strategy for Hb monitoring in patients treated with ESA and interventions to reduce Hb variability. Several factors affect Hb variability, including those that are drug related, such as pharmacokinetic parameters, clinical practice guidelines, treatment protocols, and reimbursement policies. Strategies that consider each of these factors and reduce Hb variability may be associated with improved clinical outcomes [16]. There is conflicting evidence on the effect of Hb variability on mortality with some studies demonstrating a strong association and others showing no association with mortality.

**Figure 3:**
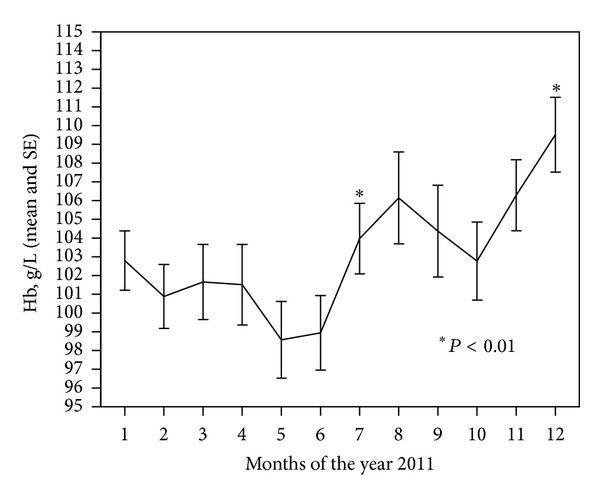
Mean hemoglobin concentrations during 2011 year. Influence of a new anemia management algorithm, certified in Lithuania August 2011.

We evaluated Hb concentrations and ESA doses in 100 patients—56 (56%) men and 44 (44%) women. The mean age of patients was 61.88 ± 14.8 years (31–84). Mean time from the start of dialysis until inclusion into the study was 4.75 ± 4.33 years. The new anemia management algorithm in Lithuania (August 2011) gave a clear rise in the Hb concentrations during the second half-year of 2011 (Figure 3). We found that Hb concentrations increased significantly with a new algorithm, though mean doses of ESA remained unchanged (11073.17 U/week versus 11425 U/week; *P* = 0.491).

**Figure 4:**
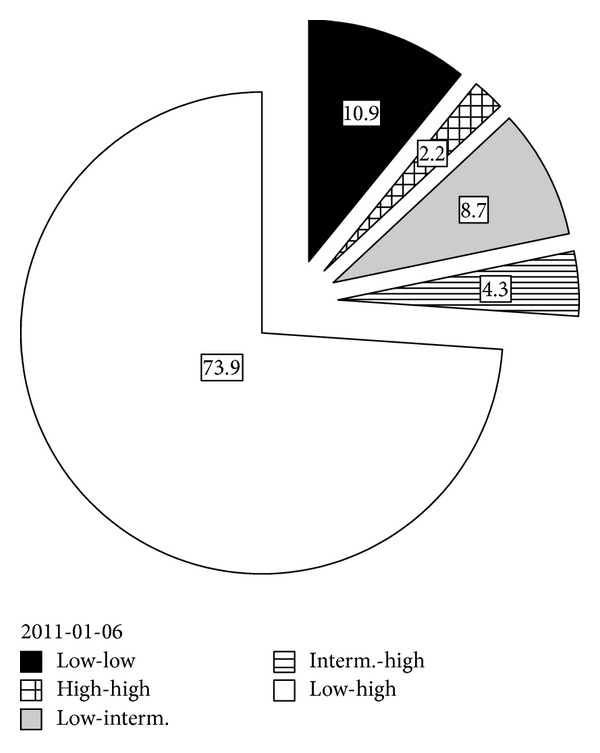
Pattern of fluctuations in hemoglobin levels during a six-month period (01/2011–06/2011) in Lithuanian hemodialysis patients, classified according to Ebben's principle (*n* = 100).

We looked in detail to each month (01/2011–06/2011) Hb concentrations and found that only 17.1% of patients during this period had Hb in the target range according to local algorithm (100–105 g/L), 50.2% of patients had Hb <100 g/L, and 32.7% had Hb >105 g/L. A big part of our patients exhibited fluctuations in the Hb levels corresponding to literature data where we found that 80–90% of ESRD patients on dialysis exhibit fluctuations in the Hb levels, known as Hb variability [16–19]. We used the six groups classification system (Ebben's principle) based on the lowest and the highest Hb categories seen during the 6-month observation period in our study and found: LL 10.9%; II 0%, HH 2.2%, LI 8.7%, IH 4.3%, and LH 73.9% of patients (Figure 4).

In the United States Renal Data System analysed by Ebben et al. [20], only 10% of patients maintained Hb levels within a single Hb category during the entire 6-month period. 29% of patients experienced Hb fluctuations between the high and target Hb groups, and 21% experienced fluctuations between the low and target Hb groups. Fluctuation across all three Hb categories during the 6-month period was observed in nearly 40% of patients [20]. We noted that none of our ESA-treated patients had Hb levels stable within the target range (100–105 g/L) over a 6-month period; 10.9% of patients had constantly low Hb concentration; 13% strayed outside their initial Hb group into the next closest group, 73.9% of the patients showed a high amplitude swing. However it is difficult to compare our data with data of other studies because a different target range of Hb concentration was used; beside, there is no single and uniformly accepted method to measure Hb variability.

**Figure 5:**
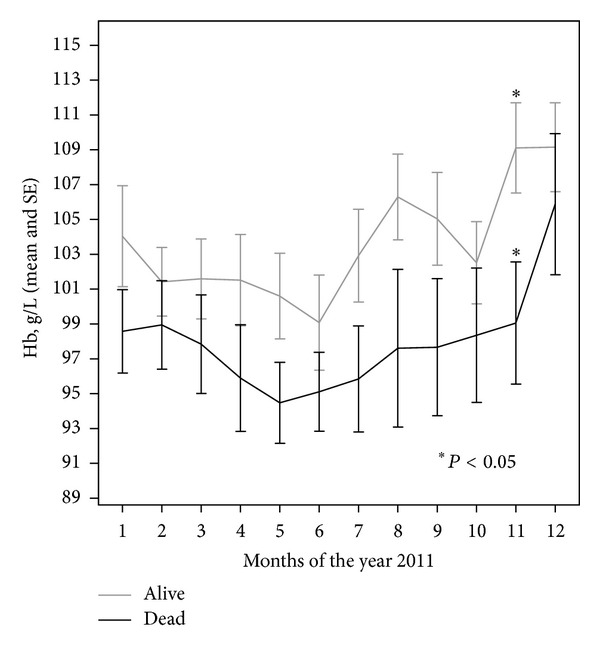
Comparison of mean hemoglobin concentrations during the year 2011 in dead and alive hemodialysis patients.

The data on the effect of Hb variability on mortality are conflicting. In our study we did not find the association between Hb variability and all-cause mortality using an adjusted Cox regression model, although the Hb concentrations of dead patients had a tendency to be lower (Figure 5) and the mean ESA doses had a tendency to be higher.

The study of Ebben et al. suggested that variability itself may not have a strong association with mortality. The key factors seem to be the number and timing of Hb values <110 g/L. Patients whose Hb levels were consistently within the target range of 110 to 125 g/L experienced the lowest mortality in their study. The longer the amount of time with Hb level <110 g/L was the greater the risk of death was noted [19]. In a study involving 34 963 HD patients Yang and colleagues reported that the risk of all-cause mortality increased proportionately with Hb variability [21]. The HR and 95% CI per 0.5 g/dL, 0.75 g/dL, 1.00 g/dL, and 1.5 g/dL increases in Hb variability were 1.15 (1.10 to 1.2), 1.24 (1.16 to 1.32), 1.33 (1.22 to 1.45), and 1.53 (1.35 to 1.75), respectively. Not all studies have demonstrated a positive association between Hb variability and death in CKD. In the study of Eckardt and colleagues [17] Hb variability was not a statistically significant factor for mortality, except in the group of patients with low amplitude fluctuations and with low Hb levels (HR 1.74, 95% CI 1.00 to 3.04) that correspond to our study data.

Our study has limitations that should be considered. The sample size was small and the data were collected retrospectively in only one dialysis center. The obtained database therefore reflects only a small sample of entire Lithuanian dialysis population. Further studies are needed to clarify the relationship between provided practices, Hb variability, and mortality.

### 3.6. Current Situation and Problems Remained

As it was mentioned before Lithuanian anemia management guidelines were revised in August 2011 and correspond to ERBP position (target Hb 100–120 g/L). The mean Hb of our HD patients was 109 ± 12.8 g/L at the end of 2011 (*P* < 0.05 as compared to 2009). 54.7% of all HD patients had Hb between 100–120 g/L, but 26.6% of them still had Hb <100 g/L. According to K/DOQI guidelines [2] target of ferritin must be 200–500 mcg/L. There were 46.4% of patients with ferritin in the target range in 2011 although the mean ferritin concentration was 375 ± 255.7 mcg/L. This percentage was similar to that in UK (45.5% in range of 200–499 mcg/L) or in Spain (45.9%) [7]. According to KDIGO [4] and K/DOQI [2] Guidelines additional intravenous iron should not routinely be administered in patients with serum ferritin levels that are consistently >500 mcg/L. According to local Lithuanian algorithm administration of iron must be interrupted when ferritin concentration exceeds 500 mcg/L; however transferrin saturation is not routinely performed in all Lithuanian HD patients. We determine a dose of iron according to ferritin concentration only, so we cannot accurately evaluate iron stores and prescribe appropriate iron dose. We are planning to continue the study of observation of renal anemia control in Lithuania, the study of Hb variability involving a larger number of patients hoping for more precise results. It is important to assess the percentage of hyporesponsiveness to ESAs in HD patients in Lithuania and to evaluate the reasons.

## 4. Conclusions

1. The history of renal anemia control in Lithuania was complicated; however, a significant improvement was achieved and 54.7% of HD patients reached the target hemoglobin.
2. The involuntary experiment with an intravenous iron occurred in Lithuania because of the economic reasons and confirmed the significant role of intravenous iron in the management of renal anemia.
3. Hemoglobin concentration below 100 g/L increased relative hospitalization risk by 1.7-fold and it was one of the most important factors influencing hospitalization rate.
4. Hemoglobin concentration below 100 g/L was associated with a 2.5-fold increased relative risk of death in Lithuanian hemodialysis patients.
5. Although hemoglobin variability was common in Lithuanian hemodialysis patients, it did not independently predict mortality.

## Figures and Tables

**Table 1:** Changes of treatment and control of renal anemia in hemodialysed patients in Lithuania.

Year	The mean Hb concentration (g/L ± SD)	Percentage of HD patients with Hb > 100 g/L	Percentage of HD patients receiving epoetin	The mean dosage of epoetin (U/week ± SD)	Percentage of HD patients receiving i/v iron
1997	92 ± 15.4	27.5	78	6071 ± 2924	7.5
1998	99 ± 15.3*	44.1*	89.5*	6537 ± 3209*	27.6*
1999	101 ± 16	52.9	92.4	7058 ± 3732	35.1
2000	104 ± 15^#^	62.9	96.1	7092 ± 3424	70.8^#^
2001	105 ± 14.7	64	94.6	9336 ± 3571^∼^	**20.9*∼***
**2002**	101 ± 14.0^∼*˚*^	51.8^∼*˚*^	88.8^∼*˚*^	7145 ± 3882^**˚**^	Only single patient
2003	100 ± 14.5	49.7	91.1	8166 ± 5525	Only single patient
2004	101 ± 13	53.6	89	8121 ± 6243	Only single patient
**2005**	105 ± 13.8^∧^	65.1^∧^	84^∧^	6768 ± 4372^∧^	86.9^∧^
2006	104 ± 13	62.4	78.8	7230 ± 4295	70.8
2007	105 ± 12.8	66.6	81.4	6716 ± 4417	85.1
2008	105 ± 13.6	66.3	76.6	6014 ± 4932	80.3
2009	105 ± 14.6	64.4	75.9	6196 ± 4414	78.2
2010	107 ± 13.6	67.8	76.6	6623 ± 4812	69.9

*P < 0.05 as compared to 1997, ^#^ *P* < 0.05 as compared to 1999, ^∼^ *P* < 0.05 as compared to 2000, °*P* < 0.05 as compared to 2001, and ^∧^ *P* < 0.05 as compared to 2004.

**Table 2:** Relationship between iron deficiency and dose of erythropoietin in the treatment of renal anemia of hemodialysed patients in 2002–2005.

Year	The mean Hb concentration (g/L ± SD)	Percentage of HD patients with ferritin <100 mcg/L	Percentage of HD patients receiving epoetin	The mean dosage of epoetin (U/week ± SD)
2002	101 ± 14.0	30.5	88.8	7145 ± 3882
2003	100 ± 14.5	49.5^∧^	91.1	8166 ± 5525^∧^
2004	101 ± 13	60.9^∧^	89	8121 ± 6243
2005	105 ± 13.8^∧^	18.5^∧^	84^∧^	6768 ± 4372^∧^

^∧^ *P* < 0.05, as compared to previous year.

**Table 3:** Relative risk of death for hemoglobin categories.

Mean hemoglobin concentration (g/L)	Relative risk	*P*	95% CI	
			Lower limit	Upper limit
100–105	1.0			
**<100**	**2.472**	**<0.001**	1.923	3.177
106–109	1.076	0.687	0.754	1.534
110–120	1.058	0.731	0.767	1.459
121–130	1.031	0.915	0.593	1.791
>130	2.356	0.063	0.953	5.822
